# Extracorporeal carbon dioxide removal with continuous renal replacement therapy. Case description and literature review

**DOI:** 10.5935/0103-507X.20200020

**Published:** 2020

**Authors:** Marta López-Sánchez, María Isabel Rubio-López

**Affiliations:** 1 Intensive Care Unit, Hospital Universitario Marqués de Valdecilla - Santander (Cantabria), Spain.

**Keywords:** ECCO_2_R, Lung transplantation, Carbon dioxide, Renal replacement therapy, ECCO_2_R, Trasplante de pulmón, Dióxido de carbono, Terapia de reemplazo renal

## Abstract

In recent years and due, in part, to technological advances, the use of extracorporeal carbon dioxide removal systems paired with the use of extracorporeal membrane oxygenation has resurfaced. However, studies are lacking that establish its indications and evidence to support its use. These systems efficiently eliminate carbon dioxide in patients with hypercapnic respiratory failure using small-bore cannula, usually double-lumen cannula with a small membrane lung surface area. Currently, we have several systems with different types of membranes and sizes. Pump-driven veno-venous systems generate fewer complications than do arteriovenous systems. Both require systemic anticoagulation. The “lung-kidney” support system, by combining a removal system with hemofiltration, simultaneously eliminates carbon dioxide and performs continuous extrarenal replacement. We describe our initial experience with a combined system for extracorporeal carbon dioxide removal-continuous extrarenal replacement in a lung transplant patients with hypercapnic respiratory failure, barotrauma and associated acute renal failure. The most important technical aspects, the effectiveness of the system for the elimination of carbon dioxide and a review of the literature are described.

## Abstract (es)

En los últimos años, y debido en parte a los avances tecnológicos, ha resurgido el uso de los sistemas de depuración extracorpórea de dióxido de carbono de manera pareja al uso de la oxigenación con membrana extracorpórea. No obstante, faltan estudios para establecer sus indicaciones y el nivel de evidencia para su uso. Estos sistemas permiten eliminar el dióxido de carbono de manera eficaz en pacientes con insuficiencia respiratoria hipercápnica con catéteres de pequeño calibre, habitualmente de doble luz y con pequeña superficie de membrana depuradora. En la actualidad disponemos de varios tipos de sistemas, con distinta versatilidad y tamaño de membrana. Los sistemas veno-venosos con bomba producen menos complicaciones que los arterio-venosos. Ambos precisan anticoagulación sistémica. El soporte “pulmón-riñón” mediante la combinación de un sistema depurador con un hemofiltro permitiría al mismo tiempo eliminar dióxido de carbono y realizar depuración extrarrenal continua. Describimos nuestra experiencia inicial con un sistema combinado de depuración extracorpórea de dióxido de carbono-depuración extrarrenal continua en un paciente con trasplante de pulmón, insuficiencia respiratoria hipercápnica, barotrauma y fallo renal agudo asociado. Se describen los aspectos técnicos más importantes, la efectividad del sistema para la eliminación de dióxido de carbono y se realiza una revisión de la literatura.

## INTRODUCTION

Carbon dioxide (CO_2_) removal systems (ECCO_2_R - *extracorporeal carbon dioxide removal*) have been utilized since the 1970s. In 1986, Gattinoni^(1)^ published a series of 43 patients with severe acute respiratory distress syndrome (ARDS) treated with an ECCO_2_R device together with mechanical ventilation (MV) with a low respiratory frequency (LFPPV - *low frequency positive-pressure ventilation*) and pressure limitation (“lung at rest”), with improvement in lung function in 78.8% of patients. In 1990, Terragni^(2)^ showed that a system including a 0.33m2 neonatal membrane lung and a hemofiltration cartridge could reduce the tidal volume (Vt) below 6 mL/kg predicted body weight, normalizing the hypercapnia generated and reducing the cytokine concentration in bronchoalveolar lavage fluid at 72 hours in 32 patients with ARDS.

Currently, these systems have become popular due to technological advances and greater knowledge of lung damage induced by mechanical ventilation (VILI - *ventilator-induced lung injury*). They are capable of efficiently removing CO_2_; therefore, they are promising for facilitating protective or ultraprotective MV in ARDS, with the hypothesis that a greater reduction in Vt and plateau pressure (Pplat) could be accompanied by a decrease in mortality, avoiding alveolar overdistention that occurs with the use of protective MV.^(3,4)^ In the recently published SUPERNOVA study, these systems were able to facilitate ultraprotective MV in patients with moderate ARDS.^(3)^

These systems, which operate with lower blood flow and membrane surface than does an ECMO (extracorporeal membrane oxygenation) system and whose function is to remove CO_2,_ have potential indications in hypercapnic patients.^(5,6)^ In patients with chronic obstructive pulmonary disease (COPD), ECCO_2_R systems avoid the need for MV and are used as alternatives to MV when noninvasive MV (NIMV) fails or for facilitating extubation. As a bridge to lung transplantation (LT), these systems are viable alternatives that improve the physical condition of patients while they wait for a compatible organ, avoiding complications arising from MV and enhancing rehabilitation, respiratory physiotherapy, nutritional status and, even mobility, which are important in these patients.^(7,8)^

Currently, we have a broad spectrum of ECCO_2_R systems, most of which are veno-venous. Some only remove CO_2_ while others escalate therapy to ECMO, and some have a membrane attached to the pump while others have an independent membrane. The addition of a CO_2_ removal membrane together with a hemofilter would maintain vascular access, increase system safety, adapt anticoagulation, combine it with continuous renal replacement techniques (CRRTs) and possibly increase the efficiency of CO_2_ lavage.^(2)^ In addition, with this “lung-kidney” support, a reduction in vasopressor requirements has been demonstrated.^(9)^

We present a case in which a combined ECCO_2_R-CRRT system was used, describing its effects and discussing the most important technical aspects.

## CASE REPORT

A 49-year-old male patient was admitted to the intensive care unit (ICU) for acute respiratory failure. The patient underwent two-lung transplantation due to idiopathic pulmonary fibrosis 2 months earlier, with grade 2 primary graft dysfunction and A2B1 acute cellular rejection. Cultures were taken, empiric antibiotic therapy was initiated, and immunosuppressive therapy was adjusted, requiring MV for global respiratory failure, with a marked reduction in respiratory compliance (11.4mL/mbar) with a plateau pressure (Pplat) of 35cmH_2_O and peak pressure (Pp) of 40cmH_2_O, with a Vt of 330mL and 20 breaths/minute. With a fraction of inspired oxygen (FiO_2_) of 0.4 and positive end-expiratory pressure (PEEP) of 5cmH_2_O, arterial gas analysis showed the following: pH, 7.11; partial pressure of carbon dioxide (PaCO_2_), 55.7mmHg; partial pressure of oxygen (PaO_2_), 113mmHg; bicarbonate, 24mmol/L; and excess of bases, -0.4mmol/L. The Vt gradually decreased to 220mL, and the respiratory rate (RR) decreased to 15 breaths/minute. PEEP was withdrawn in the presence of bilateral tension pneumothorax and Pplat flow limitations. Next, PaCO_2_ was increased to 87 mmHg with pH 7.11.

The patient also presented with acute kidney injury (AKI), with creatinine and urea levels of 0.7mg/dL and 26mg/dL, respectively, and with oliguria, bleeding from thoracic drainages, previous barotrauma and severe thrombocytopenia (46000/mm^3^). A Prismalung system was implanted using a 13.5 Fr femoral cannula. Hemodynamic monitoring was performed by intermittent invasive blood pressure measures of central venous oxygen saturation (SatvO_2_), and a negative fluid balance was monitored during therapy.

**Figure 1:**
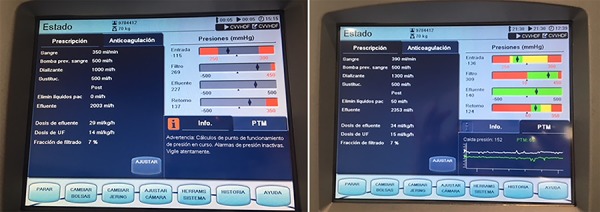
Detail of the parameters and pressures for the extracorporeal carbon dioxide removal-continuous extrarenal replacement system with a flow of 350mL/minute and with a flow of 390mL/minute (side panel).

**Figure 2:**
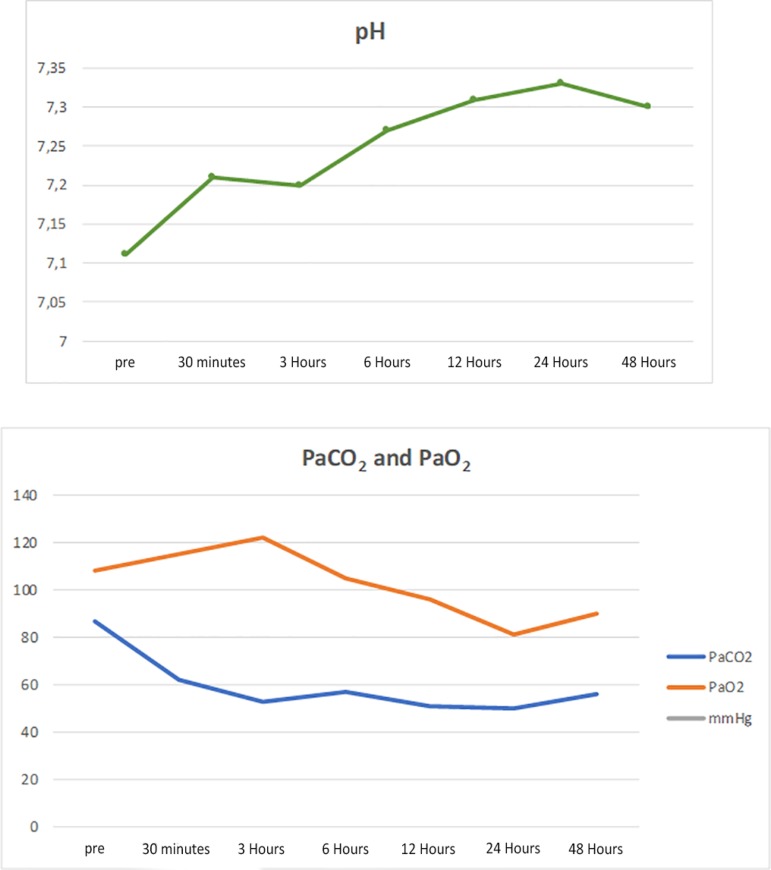
pH, PaCO_2_ and PaO_2_ curves before and after device implantation. pH - hydrogen potential; PaCO_2_ - partial pressure of carbon dioxide; PaO2 - partial pressure of oxygen.

Figure 1 shows the details of the parameters and pressures generated through the system with a blood flow of 350mL/minute. At the time of initiating therapy, PaCO_2_ decreased to 62mmHg with a pH of 7.21. The pH improved, and PaCO_2_ decreased without modifying PaO_2_ (Figure 2). After 24 hours, the flow was increased to 390mL/minute with an increase in pre-filter pressure (Figure 1) without modifying PaCO_2_.

On the first day of support, a maximum activated partial thromboplastin time (aPTT) of 1.5 was maintained for thrombocytopenia. On the second day, the minimum aPTT was 1.09, and the maximum was 1.23. After 48 hours, thrombosis within the hemofilter was present, with an aPTT ratio of 1.1, and the CO_2_ removal membrane was removed. Renal support therapy was continued, percutaneous tracheostomy was performed, and after an initial improvement, pneumonia associated with *P. aeruginosa* and MV developed. The patient died of multiorgan failure without device-related complications.

## DISCUSSION

The elimination of CO_2_ with extracorporeal systems, both through ECMO and with ECCO_2_R devices, is effective, but the latter contributes minimally to oxygenation, as they are low-flow systems. The potential indications for ECCO_2_R are included in the introduction.

The possible adjustment of MV (increasing PEEP) and increase in alveolar O_2_ pressure by reducing the alveolar CO_2_ pressure (PACO_2_) may explain the slight improvement in oxygenation. In addition, according to the hemoglobin dissociation curve, in an arteriovenous ECCO_2_R system, the oxygenating capacity is lower because only a few milliliters of O_2_ can be added to oxygenated blood; however, in the case of veno-venous systems_,_ approximately 35mL of O_2_ can be supplied (assuming venous O_2_ saturation of 75% and a hemoglobin level of 10g/dL).^(10)^

### Definition of ECCO_2_ R and the basis of its operation

ECCO_2_R systems are low-flow (250 - 1500mL/minute) partial respiratory support devices, with a smaller membrane surface (0.33 - 0.67m2, with larger surfaces used with versatile systems that allow transition to ECMO). The diffusion capacity of CO_2_ across the membrane is approximately 20 times higher than that of O_2_, and 200 - 250mL/minute of CO_2_ production in an adult can be removed with a flow of 500mL/min.^(5,10,11)^ In our case, the increase in flow from 350 to 390mL/minute increased the pre-filter pressure (Figure 1).

The main determinant of CO_2_ lavage is air flow, with a maximum of 10L/minute recommended for most devices.^(10,11)^ In our case, the increase to 12 - 15L/minute did not further reduce PaCO_2_. Regarding blood flow, in a bovine animal model, a flow between 750 - 1000mL/minute was more effective than a flow between 250 - 500mL/minute, regardless of the membrane size used, although a membrane surface of 0.8m2 was more effective than a membrane surface of 0.4m2.^(12)^ However, in a porcine venous model with veno-venous ECMO, an increase in both blood flow and air flow reduced PaCO_2_ in apneic ventilation.^(13)^

A very recent publication concluded that low-flow ECCO_2_R systems should be limited to mild respiratory acidosis or to facilitate MV in ARDS.^(14)^Table 1 shows the different membrane surfaces used in the studies published with ECCO_2_ R-CRRT combined systems.

**Table 1:** Summary of the studies with combined extracorporeal carbon dioxide removal and continuous extrarenal replacement systems

Author	Population	No. of cases	Configuration	Membrane(m_2_)	Hemofilter (m^2^)	Blood flow (mL/min)	Air flow (L/min)	Cannula (Fr)	Anticoagulation
Terragni et al.^(2)^	Humans	32	VV	0.33 Polystan	Medica D200	500	8	14 DL	aPTT ratio 1.5
Forster et al.^(9)^	Humans	10	VV	0.67	1.4 polysulfone	250 - 500	6 - 7	13 DL	aPTT at 60 sec. ACT: 120 - 200 sec
Young et al.^(16)^	Animal	9	AV	5	-	470 - 600	10	-	ACT 200 - 300 sec
Quintard et al.^(17)^	Humans	16	VV	0.65 polypropylene	1.4 polysulfone	400 - 500	10	DL: 13.5 jugular (15cm) or 13.5 femoral (24cm) or 16 femoral (27cm) DUL 13.5	aPTT 45 - 50 sec
Allardet-Servent et al.^(18)^	Humans	11	VV	0.65 polymethylpentene	1.5 polysulfone	410 ± 30	8	15.5 DL (15 and 20cm)	aPTT ratio 1.5

VV - veno-venous; DL - double-lumen cannula; aPTT - activated partial thromboplastin time; AV - arteriovenous; ACT - activated clotting time; DUL - two single-lumen cannula.

### History of the ECCO_2_ R system combined with CRRT

The “lung-kidney” support allows either only respiratory support or both types of support. Sixty percent of patients who suffer multiorgan failure and require MV also develop acute kidney failure (AKF). In these patients, fluid overload and increased alveolar permeability derived from AKF negatively affect the lungs, and in the same way, MV and biotrauma affect renal function.^(15)^

The first described ECCO_2_R-CRRT system dates back to 1992 and was an arteriovenous system in an animal model.^(16)^ Subsequently, in the 2013, Forster^(9)^ applied this therapy to 10 patients with ARDS with respiratory acidosis (mean PaCO_2_, 69mmHg) and AKF. This veno-venous system was composed of a 1.4 m2 hemofiltration membrane, a 13Fr double-lumen cannula inserted in the jugular vein and an ECCO_2_R^2^ membrane with a surface area of 0.67m2, with a blood flow of 250 - 500mL/min (mean 378mL/min) and air flow of 4 - 6L/minute (Table 1).

In 2014, Quintard^(17)^ implanted a combined system in 16 patients with respiratory acidosis (mean PaCO_2_ 77.3mmHg) and AKF. They used a 0.65m2 membrane and greater airflow (up to 10L/minute), with different cannula thicknesses and placements, either single or double lumen and with a variable gauge (13.5 - 16Fr), an aspect that possibly influenced the marked reduction in CO_2_ at 3 hours (31%) and at 6 hours (39%). There were no significant complications (Table 1).

In 2015, Allardet-Servent^(18)^ used a combined system in 11 patients with ARDS, a lung injury score (LIS) of 3 ± 0.5 and a PaO_2_/FiO_2_ ratio of 135 ± 41. A PaCO_2_ reduction of 21% was observed, allowing a decrease in Vt to 4mL/kg of predicted weight using a blood flow of 410 ± 30mL/minute with a CO_2_ removal of 83 ± 20mL/minute (Table 1).

### Technical aspects of the combined ECCO_2_ R-CRRT system

These systems require anticoagulation with sodium heparin, with monitoring of aPTT^(15-18)^ and/or activated coagulation time (ACT),^(9)^ as shown in table 1. An aPTT ratio between 1.5 - 2 is recommended, weighing the risk of hemorrhage and/or thrombosis.^(6,11)^ In our case, coagulation of the hemofilter (but not the membrane lung) occurred at 48 hours, coinciding with a low aPPT ratio (<1.5) due to the risks of anticoagulation. With these systems, the role of citrate as an alternative to sodium heparin is undetermined. In an animal model, local citrate anticoagulation was as effective as sodium heparin but did not increase CO_2_ removal and led to increased hypercalcemia and acidosis.^(19)^

In our case, the membrane lung was placed in front of the filter, as in the model described by Terragni.^(2)^ When the membrane lung is placed in this manner, the removal capacity is greater than when it is placed behind. Pre-dilution replenishment reduces blood viscosity and the concentration of coagulation factors, extending the life of the system.^(2,17,18)^

We used an AN69 hemofilter (0.9m2) with a Prismaflex v 6.0 system (Gambro, Lund, Sweden) in continuous veno-venous hemodiafiltration mode with a maximum flow rate of 390mL/minute (Figure 1). For flows > 400mL/minute, it would be advisable to use hemofilters with larger surface areas (1.5m2). Thus, in the model described by Allardet-Servent et al.^(18)^ with a diameter of 15.5Fr, flows greater than 400mL/minute were obtained with a 1.5m2 hemofilter and 0.65m2 membrane lung. With this flow, the authors obtained a CO_2_ removal similar to that achieved with an ECCO_2_R device without a hemofilter but with the same cannula diameter and similar flow.

### Complications of ECCO_2_R systems

Thrombotic complications are the most feared because they indicate systemic changes and limit treatment. In the studies cited, thrombosis of the hemofilter and another of the cannula^(18)^ are described, as is the absence of complications.^(17)^

With ECCO_2_R in general, thrombosis of the removal membrane occurs in 14 - 16.7% of cases,^(3,10,11)^ and hemorrhaging occurs in 2 - 50% of cases.^(3,10)^ Other complications include hemolysis, thrombocytopenia, hypofibrinogenemia, cannula infection, cannula loss or displacement, recirculation, air embolism, and vascular complications (limb ischemia, compartment syndrome, aneurysm, pseudoaneurysm, and hematoma), with the latter occurring with arteriovenous devices.^(3,6,10)^

## CONCLUSION

Combined extracorporeal carbon dioxide removal-continuous extrarenal replacement with a flow rate that did not reach 400mL/minute was effective for the removal of carbon dioxide but was limited by rapid thrombosis of the hemofilter.
